# Mitotic Recombination Accelerates Adaptation in the Fungus Aspergillus nidulans

**DOI:** 10.1371/journal.pgen.0030068

**Published:** 2007-04-27

**Authors:** Sijmen E Schoustra, Alfons J. M Debets, Marijke Slakhorst, Rolf F Hoekstra

**Affiliations:** Laboratory of Genetics, Wageningen University, Wageningen, the Netherlands; University of Toronto, Canada

## Abstract

Understanding the prevalence of sexual reproduction in eukaryotes is a hard problem. At least two aspects still defy a fully satisfactory explanation, the functional significance of genetic recombination and the great variation among taxa in the relative lengths of the haploid and diploid phases in the sexual cycle. We have performed an experimental study to explore the specific advantages of haploidy or diploidy in the fungus Aspergillus nidulans. Comparing the rate of adaptation to a novel environment between haploid and isogenic diploid strains over 3,000 mitotic generations, we demonstrate that diploid strains, which during the experiment have reverted to haploidy following parasexual recombination, reach the highest fitness. This is due to the accumulation of recessive deleterious mutations in diploid nuclei, some of which show their combined beneficial effect in haploid recombinants. Our findings show the adaptive significance of mitotic recombination combined with flexibility in the timing of ploidy level transition if sign epistasis is an important determinant of fitness.

## Author Summary

Sexual reproduction involves an alternation of ploidy. Haploid gametes, carrying a single set of chromosomes, fuse to form a diploid zygote with a double set of chromosomes. The gametes are formed from diploid progenitor cells by meiosis, which involves genetic recombination—the key evolutionary aspect of sexual reproduction. In this paper we show that in the fungus *A. nidulans,* during somatic growth, mitotic recombination occurs at a sufficiently high rate to allow an acceleration of the adaptation to novel environmental conditions. Because fungi (unlike animals) lack a clear soma-germline distinction, nuclei with a novel recombinant genotype in the somatic tissue (the mycelium) can give rise to progeny in the form of asexual spores. The results show that recombination at the somatic level (so-called parasexual recombination) appears to be of evolutionary relevance. This finding recalls a suggestion that was made 50 years ago by Pontecorvo, but was discredited soon afterwards.

## Introduction

Sexual cycles involve an alternation between a haploid and a diploid phase. The relative duration of both ploidy phases may differ strikingly among taxa. Extremes are, on the one hand, diploid multicellular animals and plants with haploidy restricted to the gamete stage, and on the other hand, haploid algae and fungi with diploidy restricted to the zygote stage. The great variation in predominance of the diploid state in eukaryote life cycles is not fully understood, despite a long history of theoretical [1–4] and experimental [5–9] investigation. Possible evolutionary advantages of diploidy include the masking of deleterious recessive mutations [2,4] and faster adaptation [3,8], but a higher rate of adaptation in haploids has also been found in several studies with yeast [6,9].

**Figure 1:**
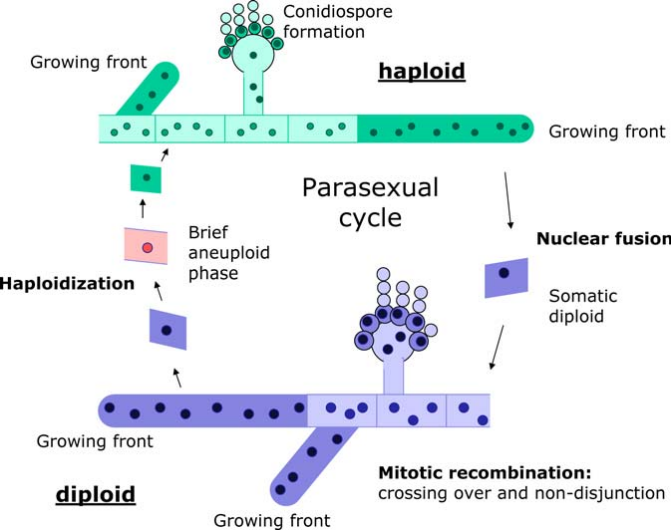
Schematic Overview of the Parasexual Cycle in the Filamentous Fungus *A. nidulans:* An Alternation between Vegetative Growth with Haploid or Diploid Nuclei The fungal mycelium consists of a network of hyphae and the formation of (a)sexual spores on the surface. Mitotic activity takes place in the apical cells at the growing front of the colony (dark shaded cells) and in sterigmata where asexual spores are formed. The parasexual cycle starts by nuclear fusion of two vegetative haploid nuclei into a vegetative diploid nucleus; this can give rise to a vegetative mycelium. Diploid mycelia have the same architecture as haploid mycelia, but contain half the number of nuclei [11,29]. Repeated nondisjunction during mitosis in a diploid nucleus can revert it to haploidy by random loss of whole chromosomes, leading to recombination at the chromosome level. Also, partially homozygous diploids can arise by nondisjunction (when diploid nuclei go through a brief 2*N*+1 aneuploid phase) and mitotic recombination [10,11,13].

The mycelial fungus and genetic model organism A. nidulans allows for facile comparisons between the advantages of haploidy and diploidy. Along with the sexual cycle it has, like many other fungi, a “parasexual cycle” [10] (Figure 1). In growing mycelia, haploid nuclei may fuse with a probability of 10^−6^ to form relatively stable vegetative diploid nuclei. Diploid nuclei can spontaneously produce diploid recombinants by mitotic crossing-over and haploid recombinants by repeated loss of whole chromosomes with a probability of 10^−3^ per mitosis [11–13]. Therefore, the ploidy level of the nuclei is polymorphic in mycelia of intermediate-to-large size. At any point in time a small fraction of its nuclei will exist in the diploid state and act as a source of recombinant haploid nuclei. If a diploid nucleus is heterozygous at some loci (because of mutations or when the mycelium is a heterokaryon), a haploid recombinant may contain novel allelic combinations. If adaptation involves “sign-epistasis”—defined as an interaction between mutations that are individually neutral or deleterious but advantageous when combined—for which there is growing empirical support [14], the vegetative diploid stage may act as an accumulator of recessive mutations of this type. Specific advantageous combinations of mutations may then appear in haploid nuclei produced by parasexual recombination. Such successful haploid segregants may then get fixed in the mycelium as the sector containing them outgrows the rest of the colony. We hypothesize that the flexibility of switching between diploidy and haploidy within the vegetative organism, as provided by the parasexual cycle, may allow particularly fast adaptation if sign epistasis is involved.

We tested this hypothesis in an experimental study of adaptation over 3,000 mitotic generations by comparing adaptation in 15 haploid and 20 homozygous diploid strains of *A. nidulans.* At the start of the experiment, all strains were genetically identical except for ploidy and three neutral genetic markers. Propagation in our experiment was exclusively vegetative by inoculating a small part of the growing front of a colony with the highest mycelial growth rate (MGR) six days after incubation onto fresh medium. Fitness is therefore defined as the MGR of a colony on the surface of a solid medium [15,16]. The strains used carry a resistance mutation to the fungicide fludioxonil causing a 50% reduction in MGR in the absence of the fungicide when compared to wild-type strains. We studied adaptive recovery during growth on fungicide-free medium.

## Results

**Figure 2:**
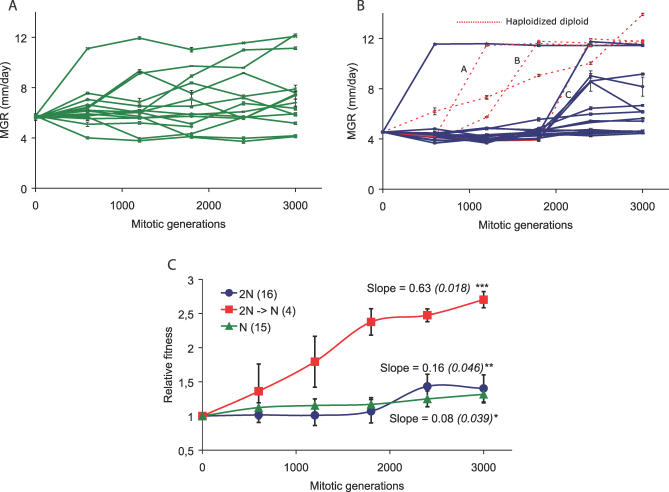
Fitness Trajectories of Evolving Strains Fitness trajectories of haploid (A) and diploid (B) strains; each line in (A) and (B) represents one single evolving strain. Haploidized diploids are indicated with a red dashed line. The mean of all strains of a condition and ploidy, averaged over all strains is shown in (C). In (C), all haploid and diploid strains were scaled relative to their haploid or diploid ancestor; diploids were scaled to the haploid ancestor upon haploidization. The mean rate of adaptation is given by the slope of the fitness trajectories and is also shown in (C). Of the 20 replicate populations that began as diploids (B), four reverted to haploidy in the course of the experiment; they are shown with a red dashed line; their mean trajectory is shown separately in (C). Error bars represent the standard error of the mean. All mean slopes (C) are higher than zero (*t*-tests; * *t*_14_ = 2.15, *p* = 0.049; ** *t*_15_ = 3.50, *p* = 0.0032; *** *t*_3_ = 34.6, *p* < 0.0001).

Figure 2A and 2B show the fitness trajectories of all evolving strains per ploidy level. The rate of adaptation of individual populations is estimated by the slope of the fitness trajectory; the mean of these fitness trajectories is shown in Figure 2C. Fitness improvement was caused by compensatory mutations rather than by reversion to fungicide sensitivity, since all strains retained their resistance during the evolution experiment. Haploid strains show a fast response; most variation among the 15 evolving strains is reached within the first 1,500 mitotic generations. Change in fitness occurs gradually and, when compared to the diploid strains, in relatively small steps. By using benomyl and by measuring spore diameter (see Materials and Methods), we found that all strains that began as a haploid remained haploid during the 3,000 mitotic generations. Diploid strains show a delayed response; most variation among the 20 evolving strains begins to arise after 1,500 mitotic generations. Change in fitness occurs in relatively large steps as compared to the haploid strains. Of the 20 initially diploid strains, four reverted to haploidy in the course of the experiment. Three of these (marked A, B, and C in Figure 2B) showed an instantaneous and dramatic increase in fitness upon haploidization. In one case, fitness improvement was more gradual and continued after haploidization. In all groups of strains the mean mycelial growth rate increased over the course of the experiment (*t*-tests, haploids: *t*_14_ = 2.35, *p* = 0.034; diploids that remained diploid: *t*_15_ = 2.94, *p* = 0.010; haploidized diploids: *t*_3_ = 28.7, *p* < 0.0001). The four haploidized strains had a significantly higher mean rate of adaptation (Figure 2C) than the strains remaining haploid or diploid. Strains that started and remained haploid did not differ in mean rate of adaptation from strains that remained diploid (ANOVA, *F*_2,32_ = 19.6, *p* < 0.0001 and post–hoc testing using the Tukey-Kramer method, α = 0.05).

**Figure 3:**
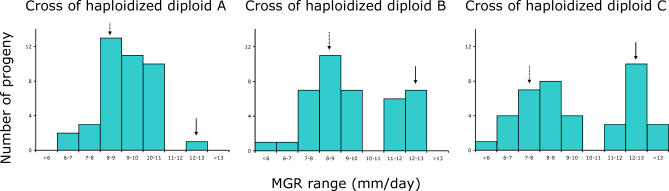
Results from Crosses between Spontaneously Haploidized Diploid Strains and the Haploid Ancestor MGR of progeny of a sexual cross of three spontaneously haploidized diploid strains (A, B, and C; see Figure 2B) and the haploid ancestor. For each cross, 40 progeny were analyzed. The solid arrow indicates the MGR class containing the evolved haploidized parent, the dashed arrow indicates the MGR class of the haploid nonevolved ancestral strain (WG631). The variety of MGRs found among the progeny and the absence of clear phenotypic classes indicates the presence of multiple adaptive mutations. Most combinations of these mutations give rise to a MGR intermediate between the values of the two parents of the cross. In all crosses considerable genetic variation in MGR exists among the 40 progeny (ANOVA data: A, *F*_39,163_ = 12.43, *p* < 0.0001; B, *F*_39,195_ = 35.09, *p* < 0.0001; C, *F*_39,192_ = 63.45, *p* < 0.0001). We compared the MGR of the progeny with the lowest MGR of each cross with the MGR of the ancestral stain (WG561 and WG615) and found that for two crosses this MGR is significantly lower than the MGR of the ancestor (A, *t*_16_ = 1.04, *p* = 0.31; B, *t*_16_ = 4.77, *p* = 0.00020; C, *t*_13_ = 6.57, *p* < 0.0001).

**Figure 4:**
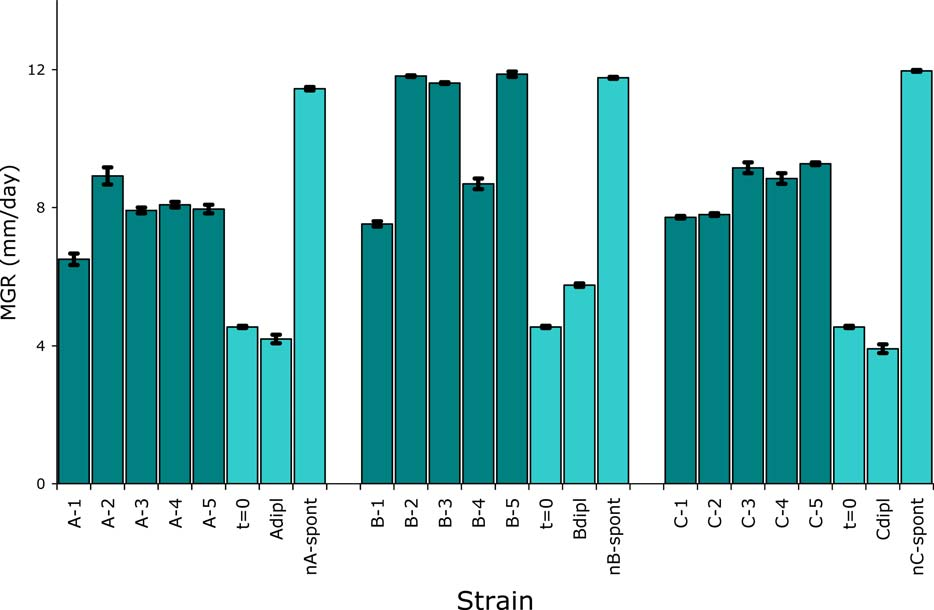
Forced Haploidization of Diploid Strains that Later Spontaneously Produced Haploid Segregants MGR of five forced haploid segregants for each of the three diploid strains that 240 generations later spontaneously gave rise to a high-fitness haploid segregant (marked A, B, and C; see Figures 2B and 3), their MGR at the start of the experiment, the MGR of the diploid evolved ancestor and the spontaneously segregated haploid. From 40 segregants, five were analyzed; they were selected on the basis of apparent vigorous growth. Error bars show the standard error of the mean of two replicates. For each diploid strain, there was significant variation in MGR among the forced haploid segregants (ANOVAs: A, *F*_4,5_ = 54.50, *p* = 0.0010; B, *F*_4,5_ = 564.0, *p* <0.0001; C, *F*_4,5_ = 46.29, *p* = 0.0003).

We further analyzed the four haploid strains with a diploid history in order to understand their remarkably fast adaptation. First, diploids were constructed combining evolved and ancestral haploid strains [12]. These heterozygous diploids expressed the non-adapted phenotype, demonstrating that the adaptive mutations are recessive. Second, the three haploid recombinants that made the largest jump in MGR upon haploidization (marked A, B, and C in Figure 2B) were crossed with a haploid ancestor. From each cross, the MGR on fungicide-free medium of 40 progeny was measured (Figure 3). The large genetic variance in MGR among the progeny and the absence of a clear segregation pattern into a limited number of phenotypic classes points to the involvement of multiple mutations in the successful spontaneous haploid revertants. The occurrence of progeny with a fitness lower than that of the nonevolved ancestor suggests that some of the mutations that have accumulated in the diploid phase during the experiment are deleterious by themselves. Third, the three diploids that showed a dramatic fitness increase upon haploidization were retrieved from the −80^ °^C stock 240 generations prior to their spontaneous haploidization, and haploidization was induced [12]. For these diploids, the MGR of five analyzed haploid segregants varied in between that of the diploid strain before spontaneous haploidization and the spontaneous haploid segregant (see Figure 4). This indicates the presence of recessive mutations that were adaptive either alone or in combination, but also indicates that the unique combinations of mutations later found in the haploid recombinants were not present yet. It also confirms our earlier conclusion that the large fitness increase of the haploid recombinants was due to a beneficial combination of multiple mutations.

Among the 16 diploid strains that remained diploid during the experiment, we found one strain in which the MGR had increased by a factor of 2.5 after 600 mitotic generations, reaching about the same level as that of the haploid recombinants (see Figure 2B). After induced haploidization of this evolved diploid strain, all 20 analyzed haploid segregants showed the same high MGR as their diploid progenitor. We performed a dominance test by combining one of these haploid segregants and a nonevolved fungicide resistant haploid strain (WG631). The resulting diploid did not show the same elevated MGR. These observations indicate that this particular diploid strain had become homozygous for a part of the genome (loss of heterozygosity), most likely by mitotic crossing-over or nondisjunction, that carried a recessive adaptive mutation with large effect, or several linked recessive mutations. The diploid strains that show a fitness increase towards the end of the evolution experiment could in a similar way have partially homozygous genomes due to mitotic recombination in the parasexual cycle, allowing expression of (combinations of) recessive adaptive mutations.

## Discussion

Our experimental results show that the shuttling between ploidy levels during vegetative growth enhances adaptation. We have demonstrated that evolved populations that started as diploid but reverted to haploidy have a higher rate of adaptation than populations that began and remained haploid or diploid*.* The haploids with diploid history first benefited from the diploid state, during which recessive mutations could accumulate irrespective of their fitness effect in haploid state. During haploidization, high-fitness recombinants were produced, in all likelihood containing combinations of interacting recessive mutations—otherwise the haploid-at-all-times strains would have adapted equally well or better. The data suggest an important role for sign epistasis [14], because some of the offspring from the crosses between the evolved haploid recombinants and the haploid ancestor had lower fitness than both parents. The occurrence of deleterious mutations is further indicated by the fact that some haploid strains evolved to a lower MGR. We think this is due to genetic drift caused by our transfer regime. In several cases there were no visible superior sectors from which the strain could be transferred, so occasionally mycelium containing a mutation with a deleterious effect on growth may have been transferred.

The findings of this study shed new light on the evolutionary role of the parasexual cycle in fungi (Figure 1). After its discovery in A. nidulans by Pontecorvo [17], the usefulness of the parasexual cycle for genetic analysis was quickly recognized, and its role was initially seen as an alternative for sexual recombination [18]. Traditionally, the starting point of the parasexual cycle is considered to be the formation by anastomosis of a heterokaryon from two different haploid mycelia. Clearly, when two different nuclear genotypes occur in a common cytoplasm, recombination by a parasexual process can generate substantial genetic variation and can serve as an alternative for sexual recombination [17]. However, heterokaryons are rare in nature due to the widespread occurrence of somatic incompatibility [19]; this has led to skepticism about the evolutionary role of the parasexual processes [20,21]. We believe that our results justify a resurrection of the view that the parasexual cycle has an important evolutionary role in fungi [18], because we show that in initially homozygous diploids sufficient genetic variation is generated by mutation to make genetic recombination effective. The important evolutionary significance of parasexual (mitotic) recombination is that it allows the organism to combine specific advantages of both ploidy levels at the somatic level. Diploid nuclei may accumulate recessive mutations that can be recombined and tested in haploid nuclei. In this way the vegetative organism may undergo genetic adaptation, resulting in the production of better-adapted spores.

Finally, we believe that the relevance of our findings is not restricted to ascomycetous fungi, but applies more generally to genetic systems that are characterized by alternation of extended haploid and diploid somatic growth, such as in basidiomycetes, including yeast, and in many algae and mosses. The parasexual cycle occurs naturally in fungi but also in distantly related oomycetes [22] and there are indications of very similar processes in human pathogens such as Cryptococcus neoformans [23] and Candida albicans [24]. In all these systems recessive mutations may accumulate in the diploid phase followed by the segregation and selection of successful haploid recombinants that may clonally spread. Even more generally, parasexual recombination at the somatic level appears to be one of the mechanisms by which competition between cells within a multicellular individual may have evolutionary significance. Within an individual soma, cells (or nuclei) may differ at the genetic level due to the switching between haploidy and diploidy or to mitotic recombination causing loss of heterozygosity [25] or at the phenotypic level due to, for example, epigenetic modification or cell-cycle position [26]. In all these cases, within-individual competition between variants may result in differential clonal outgrowth, affecting the fitness of the organism as a whole and so indirectly its reproductive success.

## Materials and Methods

### Strains.

The A. nidulans strains used in this study were isogenic and derived from the original Glasgow strain collection [11]. From strain WG562 (*lys*B5), a spontaneous mutant resistant to the fungicide fludioxonil (Novartis; 0.2 ppm) was isolated (WG561; *lys*B5; *fld*A1: resistance to fludioxonil) [16]. Neutral genetic markers were introduced into the resistant mutant WG561 to construct WG615 (*w*A3; *fld*A1; *pyro*A4); WG561 and WG615 were used to construct the fungicide-resistant diploid strain WG561//615. WG631 (*y*A2; *pro*A2; *fld*A1) was used in a cross to analyze the number of adaptive mutations and to assess dominance of adaptive mutations.

### Media and culturing.

Strains were cultured on solid Minimal Medium (MM) [11] supplemented with lysine (2.0 mmol/l), pyridoxin (0.1 mg/l) and proline (2.0 mmol/l) where needed. Strains were always incubated at 37 ^°^C. Whether the evolved populations retained their resistance to fludioxonil was assayed by comparing the MGR of evolved and nonevolved populations on MM with fludioxonil (Novartis; 0.2 ppm). Haploidization of diploids was induced [12] using 1.7 ppm of benomyl in Complete Medium [11]. Diploids (before and after adaptation) were distinguished from haploids and the moment at which spontaneous haploidization had occurred was assessed by using Complete Medium with benomyl and by using a Coulter counter to measure the diameter of asexual spores, diploids having larger spores than haploids [11]. Crosses were performed as described by Pontecorvo et al. [10].

### Fitness assays.

We defined fitness as the MGR of fungal mycelium [12,15]. After 6 d of growth, we determined the MGR by averaging the colony diameters as measured in two randomly chosen perpendicular directions. The MGR was expressed in mm/d or made relative to the MGR of the nonevolved haploid (WG561 and WG651) or diploid ancestor (WG561//651) that founded the evolution experiment.

Due to physiological differences between haploids and diploids, the fitness in terms of MGR of isogenic haploid and diploid counterparts is not identical; the ancestral haploid strain grows 33 mm in 6 d, the diploid counterpart 27 mm in the same amount of time. To check whether the differences found between adaptation in haploid and diploid strains are affected by these physiological differences, we also scaled the rates of adaptation during the evolution experiment by expressing the MGR relative to the initial value of the ancestral haploid or diploid strain (unpublished data). The MGR of diploid strains was expressed relative to the haploid ancestor after the haploidization event. In this comparison, the difference found between the four haploidized strains and the haploid-at-all-times and diploid-at-all-times strains remains highly significant (*t*-tests; *p* < 0.0001). The MGR of WG561 and WG615 is not different (*t*-test, *t*_8_ = 0.367; *p* = 0.72).

### Evolution experiment.

For this experiment, eight haploid strains were founded from WG561, seven haploid strains from WG651, and twenty diploid strains from WG561//615. All strains used carry a resistance to the fungicide fludioxonil (*fld*A1), resulting in a lowered fitness when compared to wild-type lab strains growing on funigcide-free medium, due to costs of around 50% associated with the resistance [16,27]. Adaptive recovery on solid medium without fungicide is measured by monitoring the MGR on the surface of a Petri dish. All strains evolved independently. After every 6 d of incubation at 37 ^°^C and 1 d at 4 ^°^C the part of the growing front with the highest MGR was identified. From here, a small piece of mycelium containing between 10,000 and 40,000 (nearly) genetically identical nuclei was transferred to fresh medium. The total experiment comprised 25 transfers, with about 120 mitotic generations between each transfer (estimation based both on nuclear division time and on the position of nuclei in the mycelium combined with growth characteristics of the fungus) [16,28]. At every transfer, samples of all populations were stored in a nonevolving state (at −80 ^°^C). After 25 transfers the MGR was measured under standardized conditions of samples from the frozen stocks from every fifth transfer of all strains with three replicates for each time point. The rate of adaptation was computed as the slope of the fitness trajectory [9]. Every five transfers, the ploidy of all diploid-derived strains was assessed.

## Abbreviations

MGR: mycelial growth rate
